# EMG-controlled knee orthosis lowers effort in sit-to-stand

**DOI:** 10.3389/frobt.2025.1732294

**Published:** 2026-01-08

**Authors:** Marc-Anton Scheidl, Kristin Schuh, Marek Sierotowicz, Marcel Betsch, Claudio Castellini

**Affiliations:** 1 Assistive Intelligent Robotics Lab, AIBE, Friedrich-Alexander Universität, Erlangen, Germany; 2 Orthopaedics Clinic, Universitätsklinikum Erlangen, Erlangen, Germany; 3 Institute of Robotics and Mechatronics, German Aerospace Center (DLR), Oberpfaffenhofen, Germany

**Keywords:** EMG control, human-robot interaction, impedance control, intelligent orthotics, knee exoskeleton, rehabilitation robotics, sit-to-stand

## Abstract

**Objective:**

Pilot study with ten healthy adults, testing whether a lightweight, low-cost knee orthosis equipped with EMG-driven impedance control reduces quadriceps muscle effort during the sit-to-stand (STS) transition.

**Methods:**

Ten able-bodied adults performed 15 paced STS repetitions under three conditions: without orthosis (No-Ortho), orthosis worn unpowered (Ortho-OFF; friction-compensated), and orthosis actively powered (Ortho-ON). Surface electromyography (EMG) was recorded using 8-channel thigh bracelets on both legs. EMG signals from the braced leg were processed using ridge regression and slew-rate limiting to generate a normalized control signal that dynamically scales knee stiffness while maintaining constant damping. Median values and trial-to-trial variance of the average rectified EMG (ARV) were analyzed across four distinct movement phases (SIT, UP, STAND, DOWN) using linear mixed-effects models with log-transformed data and Bonferroni-adjusted planned contrasts.

**Results:**

Powered assistance significantly reduced median bilateral ARV by 11% during the UP phase and 15% during the DOWN phase $\left(p_{\mathrm{adj}}<0.001\right)$ , with greater reductions (up to 21%) observed on the braced limb. Variance in muscle activation decreased substantially (by up to 44%) on the braced leg during the DOWN phase, suggesting more repeatable activation patterns and neuromuscular consistency across trials. No significant compensatory activation was observed in the contralateral limb. Additionally, within-session adaptation trends were observed as participants progressively increased preparatory torque during the SIT phase, while UP-phase ARV trended downward.

**Conclusion:**

A lightweight, affordable knee orthosis employing a rapid ( ≈ 10 s), minimally calibrated EMG-driven impedance controller effectively reduces quadriceps muscle activation during STS without compromising natural movement coordination. Torque capacity limitations (16 Nm) may limit effectiveness for heavier users, and further research is needed to evaluate kinematic fidelity fully.

## 1 Introduction

### 1.1 Clinical motivation

Sit-to-stand (STS) transfers are a fundamental activity of daily living, but become markedly challenging after total knee arthroplasty (TKA). Although TKA effectively relieves pain, many patients exhibit persistent quadriceps weakness and altered biomechanics, which unloads the operated limb and over-reliance on the contralateral side (Mizner and Snyder-Mackler, 2005). Similar mobility limitations and widespread use of lower-limb orthoses are reported in neurological and age-related conditions such as hemiplegia, cerebral palsy, diplegia, and frailty (Dereshgi et al., 2021; Balkman et al., 2022). Early restoration of symmetric STS is therefore a primary goal of rehabilitation.

### 1.2 Passive and active knee orthoses

Post-operative rigid braces stabilize the joint but offer no active torque (Barrera Sánchez et al., 2022). Users therefore adopt compensatory strategies such as trunk flexion or arm push-off (Kralj et al., 1990; Kotake et al., 1993). Active knee orthoses (AKOs) have the ability to augment knee extension during high-torque tasks (Kim et al., 2015; Shepherd and Rouse, 2017; Zhang et al., 2021). Typical STS moments of ≈0.4 - $0.9\;Nm\;kg^{-1}$ (Sibella et al., 2003; Kotake et al., 1993) often exceed what compact actuators can deliver continuously, so intelligent control is essential. Experimental studies confirm meaningful off-loading: Choi *et al.* reported a 19% quadriceps average rectified EMG value (ARV) reduction when torque assistance was triggered at four tested time points (Choi et al., 2021); a self-aligning rigid AKO reduced peak electromyography (EMG) by 29% in a post-stroke case (Sarkisian et al., 2022). Nonetheless, most evaluations involved healthy participants, bilateral or tethered prototypes, and rarely analyzed variability or user adaptation to the workload.

### 1.3 EMG-driven impedance control

Impedance control modulates stiffness and damping online (Huo et al., 2022; Liu et al., 2017; Villa-Parra et al., 2017). When gains scale with real-time EMG, the assistance becomes effort-proportional (Karavas et al., 2015). Choi et al. (2021) reported ≈19% reductions in quadriceps ARV during step-up and STS in healthy individuals using EMG-triggered knee assistance, while Sarkisian et al. (2022) demonstrated ≈29% peak EMG reductions and improved comfort in a post-stroke case with a self-aligning powered knee orthosis. Most recently, Gunnell et al. (2025) showed that an EMG-controlled powered knee exoskeleton reduced peak quadriceps EMG by ≈32% and increased affected-side knee torque by ≈59% in stroke survivors when providing up to $0.5\;Nm\cdot kg^{-1}$ of assistive torque. Together, these findings establish proportional EMG-based impedance control as a promising strategy for knee support during STS. We adopt this paradigm to deliver effort-proportional stiffness without mode switching.

### 1.4 Neuromuscular adaptation and usability

Repeated-measures training with a powered exoskeleton resulted in progressive decreases in EMG over 10–15 sessions, indicating motor adaptation (Kim et al., 2021). Short familiarity protocols reduced NASA-TLX workload to 34 and increased System Usability Scale scores by more than 30% among first-time users compared to untrained users (Lau and Mombaur, 2022). Acceptance studies emphasize an unobtrusive design to avoid abandonment among older adults (Shore et al., 2022).

### 1.5 Research gap

Bespoke, high-end exoskeletons or bulky do-it-yourself (DIY) solutions dominate current evidence. From a translational perspective, many powered knee orthoses remain prohibitively expensive and bulky, relying on custom frames and specialized actuation hardware. In contrast, retrofitting CE-certified postoperative braces with off-the-shelf actuators promises lighter, modular, and more affordable devices at the cost of reduced torque capacity. Whereas Gunnell et al. (2025) focused on high-torque assistance in stroke survivors using laboratory-grade hardware and high-fidelity EMG, we investigate whether a compact, retrofitted brace with lower torque capacity $\left(\approx 0.23\;Nm\cdot \;kg^{-1}\right)$ and consumer-grade EMG can still provide meaningful unloading in healthy users during STS. We thus view our work as complementary, targeting low-cost hardware and minimal calibration as prerequisites for future translational studies. Using ARV features as a proxy for muscle demand, we report a pilot study in healthy subjects to determine whether a budget-friendly solution reduces effort while largely preserving natural STS kinematics. Quantitative gait and joint-angle analyses to confirm kinematic fidelity will be addressed in future work.

The present work addresses this gap with the following contributions:

- We retrofit a compact, CE-certified postoperative knee brace with low-cost off-the-shelf actuators and dry-electrode EMG bracelets, yielding a back-drivable, ≈1.1 kg active knee orthosis that requires only a brief (≈10 s) per-user calibration.
- We implement an EMG-driven impedance controller that scales knee stiffness proportionally to a ridge-regressed EMG envelope, thereby delivering effort-proportional assistance without explicit phase-dependent mode switching.
- In a paced STS paradigm with healthy adults, we quantify not only median reductions in quadriceps activation but also changes in trial-to-trial variance across four biomechanically defined movement phases, using linear mixed-effects models with log-transformed ARV.
- We analyse within-session adaptation of both muscle activation and orthosis torque utilisation, demonstrating that participants rapidly learn to exploit the available assistance without provoking contralateral compensation.

Together, these findings demonstrate that a budget-conscious, minimally calibrated EMG-impedance controller can meaningfully unload knee extensors during STS while preserving natural coordination, thereby motivating future clinical studies in postoperative and neurologically impaired populations.

## 2 Materials and methods

### 2.1 Motorized orthosis

**FIGURE 1:**
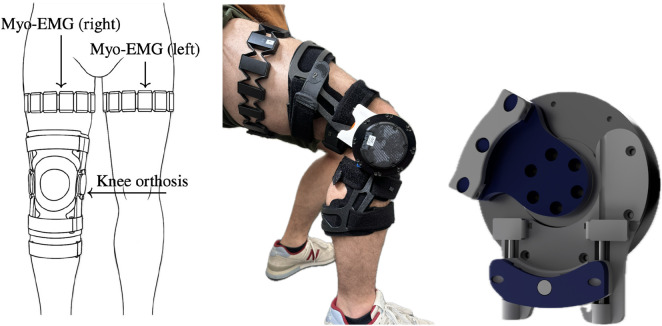
(left) Schematic of EMG bracelet and knee orthosis placement on both thighs. (middle) Experimental sit-to-stand setup with the active knee orthosis worn on the right leg. EMG bracelet is rotated by 90° for visibility. (right) Custom 3D-printed motor-brace mount connecting the AK80-9 actuator to the polycentric knee joint of the orthosis.

Our back-drivable active knee orthosis (Figure 1 center) is based on a modified GENUDYN®CI STEP THRU knee orthosis [Sporlastic (2023), Nürtingen] retrofitted with an AK80-9 brushless actuator [gear ratio 9:1, CubeMars (2023), Nanchang]. An MD80 v2.1 controller [MABRobotics (2025), Poznań], capable of 18 Nm peak and 9 Nm continuous torque, provided position, velocity, and impedance control via integrated encoders. The on board controller firmware limits peak torque to 16 Nm . In the unpowered state, the AK80-9 exhibits a measured backdrive torque of approximately 0.5 Nm CubeMars (2023). When operated in the MD80 controller’s built-in transparency mode, our measurements confirmed a mean residual reflected friction of below 0.05±0.03 Nm , ensuring effectively back-drivable behavior. The total device mass, including the actuator, brace, and custom mount, was mass≈1.1 kg , equally shared between the M-size orthosis (580 g) and motor + brace (550 g) . This is comparable to lightweight tethered knee exosuits with offboard actuators ( ≈1.1−1.7 kg on-body mass, actuators not included) (Witte et al., 2017; Park et al., 2020; Yu et al., 2022) and substantially lighter than high-torque portable knee exoskeletons ( ≈3.6 kg including electronics) by Gunnell et al. (2025). The bill-of-materials cost of the prototype is on the order of ≈1500 € excluding labour (orthosis: ≈800 €; motor + driver ≈700 €), and assembly can be completed within less than 30 min using standard workshop tools. Although this still requires specialized actuators, it is substantially lower in both cost and complexity than bespoke multi-DoF exoskeletons reported in the literature, which typically rely on custom frames and actuators (Shepherd and Rouse, 2017; Zhang et al., 2021; Sarkisian et al., 2020). Typical peak knee extension moments during STS range approximately from $0.4\text{–}1.5\;Nm\cdot \;kg^{-1}$ , depending on task setup and population (Kotake et al., 1993; Sibella et al., 2003). With a 16 Nm torque ceiling and our cohort’s mean body mass of 70.8 kg , our device’s nominal assistance $\left(\tau_{\mathrm{norm}}=0.23\pm 0.04\;Nm\cdot \;kg^{-1}\right)$ , spans roughly 15−57% of reported STS moments, depending on the reference value within this broad range.

For heavier users or faster, more explosive transfers, this torque ceiling will be reached earlier, limiting the achievable unloading. The custom 3D-printed brace (see Figure 1 right) connects the actuator via a linear push-rod, accommodating the orthosis’s polycentric knee joint and preserving natural kinematics. The source files can be downloaded from Scheidl et al. (2024). The system was operated using a Raspberry Pi 4B with headless Debian-based software (Raspbian OS, Raspberry Pi Ltd, 2025), communicating wirelessly via UDP with our external signal-processing pipeline.

### 2.2 EMG acquisition, preprocessing and control-design

#### 2.2.1 Acquisition: hardware and sensor positioning

Surface electromyography (sEMG) was acquired using two Thalmic Labs (2023) MYO bracelets, each providing eight dry-electrode channels sampled at 200 Hz. Chosen for practical considerations such as affordability, ease of use, and suitability for rapid prototyping (Mendez et al., 2017; Bangaru et al., 2020; Cognolato et al., 2018), these devices reliably capture global muscle activity for simple, single-degree-of-freedom tasks like STS and have been validated in various prosthetic user studies (Brusamento et al., 2020; Boschmann et al., 2021; Fajardo et al., 2021). The bracelets were positioned circumferentially on the proximal thigh, approximately 30% along the line from the anterior-superior iliac spine (ASIS) to the superior patellar margin (see Figure 1 left). In this configuration, each bracelet extended from the medial to the lateral aspect of the anterior thigh, i.e., from the “inner” to the “outer” quadriceps region. This arrangement was chosen to prioritize coverage of the knee extensor musculature (vastus medialis, rectus femoris, vastus lateralis), while accepting some inevitable cross-talk from neighbouring musculature and hip flexors. In particular, the biarticular rectus femoris spans both the hip and the knee, so hip flexion and extension can modulate the recorded activity. Additional variations in electrode positioning and spacing due to thigh circumference and femoral length differences among participants were given. Although this placement slightly deviated from SENIAM recommendations to avoid interference from the orthosis shell, capturing signals from the upper quadriceps extensor muscles (vastus medialis, rectus femoris, and vastus lateralis) was successful Barbero et al. (2012). The ridge regression approach employed here offers inherent robustness to shifts in electrode placement or anatomical variations, enabling rapid, user-specific calibration in seconds without the need for precise muscle targeting, facilitating a “plug-and-play”, layperson-friendly application. Dry electrodes are inherently more sensitive to sweat, skin-electrode impedance changes, and minor bracelet shifts. We mitigated these factors by normalizing EMG to an MVC-based calibration and by using ridge regression, which distributes weights across channels and is less affected by local artefacts or cross-talk. Short calibration times and the possibility to recalibrate within seconds further reduce the practical impact of slow drift.

#### 2.2.2 IMU acquisition, calibration, and forward kinematics

Body-worn inertial measurement units (IMUs, BNO08X, ≈200 Hz ) were mounted on trunk and lower-limb segments to record segment orientations for offline phase segmentation. Quaternion-based forward kinematics with anthropometric segment parameters (Drillis et al., 1964) provided estimates the Center of Mass (CoM) relative motion, particularly the onset of vertical movement, and allowed robust detection of STS phase transitions. The IMU data were not used for real-time control.

#### 2.2.3 Preprocessing: filtering and intent detection

**FIGURE 2:**
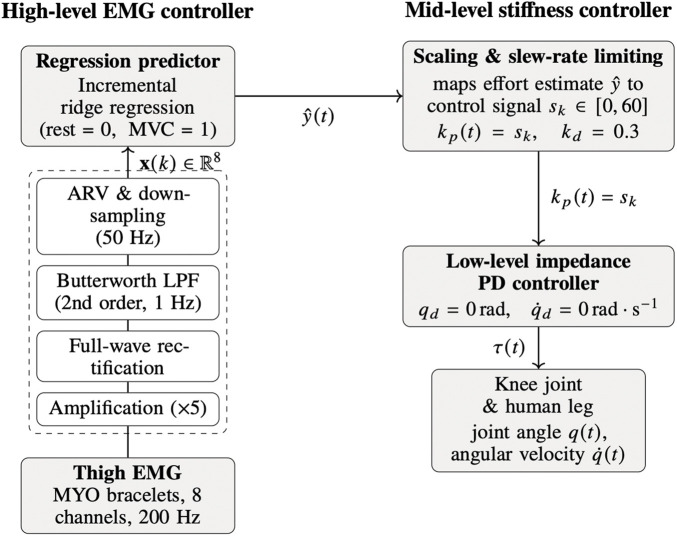
EMG processing and EMG-based stiffness control for the active knee orthosis.

Our full control pipeline can be seen in Figure 2. For our control, the EMG bracelet on the instrumented (right) thigh was used. EMG from the contralateral leg was recorded for offline analysis of symmetry and contralateral activation, but did not influence the control signal. Each EMG channel of the right-leg bracelet underwent full-wave rectification and was then processed through a zero-phase, second-order Butterworth low-pass filter (cut-off frequency 1 Hz) to extract the linear envelope, referred to as the average rectified value (ARV). This low cut-off smooths potentially noisy signals from the employed dry-electrodes and stabilizes the ridge-regression mapping at the cost of a modest temporal lag on the order of a few hundred milliseconds. Analytically, combining the 1 Hz envelope with the asymmetric slew-rate limiter yields an effective rise time of approximately 250–300 ms from a step-like EMG increase at rest to reaching the 16 Nm motor torque limit at 90° knee flexion, which is well within typical real-time budgets (<300 ms) reported for myoelectric control (Smith et al., 2011; Farrell and Weir, 2007; Igual et al., 2019; Tam et al., 2021; Attig et al., 2017). The envelope signals were subsequently downsampled to 50 Hz to reduce computational load and concatenated into an eight-dimensional feature vector $x\in R^{8}$ representing the global activation pattern of the braced thigh. A ridge regression estimator mapped this vector to a scalar effort estimate $\hat{y}=f\left(x\right)$ (Equation 1), which was used to modulate stiffness. Thus, control relied on a global activation measure for the assisted thigh rather than a single muscle channel. It was trained online over a 10-s calibration period: 5 s of rest (baseline, y=0 ) followed by 5 s of leg extension with maximum voluntary contraction (MVC, y=1 ) while seated. Post calibration, EMG vectors x were normalized to the range [0,1], and model weights (w) were fixed for the remainder of the session.

$$\hat{y}=w^{⊤}x+b,\;\underset{w,b}{\min}\underset{i}{\sum }\|{\hat{y}}_{i}-y_{i}{\|}^{2}+\lambda \|w{\|}^{2},$$ (1)

Because the regression target during calibration is a piecewise constant label (rest versus MVC) rather than a continuous kinematic variable, standard trajectory-tracking metrics such as RMSE versus joint angle are not directly informative. Instead, we verified controller adequacy by confirming that the estimated control signal reliably distinguished between rest and strong activation during the calibration window and produced stable stiffness modulation during STS across all participants.

#### 2.2.4 Control design: scaling and slew-rate limiting

The predicted EMG output $\hat{y}$ was linearly mapped to a proportional stiffness gain $k_{p}\in \left[0,60\right]\;Nm\cdot rad^{-1}$ . This mapping was heuristically determined during preliminary tests, ensuring sufficient responsiveness and comfort. At approximately 90° knee flexion, a maximum theoretical torque of about $60\;Nm\cdot rad^{-1}\times \frac{\pi }{2}\;rad\approx 94\;Nm$ could be commanded initially, exceeding the motor’s actual peak capability ≈16 Nm . Practically, this stiffness ensured that minimal EMG activity at seated positions produced near-maximal actuator response, facilitating initial torque assistance. This approach is designed to facilitate high responsiveness during early motion phases and increasingly required active muscle engagement at later stages of extension, leveling and equalizing muscle activation across the full ROM. To mitigate abrupt transients induced by residual noise, the control signal underwent asymmetric first-order slew-rate limiting Chesler and Durfee (1997). Here in Equation 2, $s_{k}$ denotes the filtered scalar control signal that is subsequently mapped to the proportional stiffness gain $\left(k_{p}=s_{k}\right)$ .

$$s_{k}=\left\{\begin{array}{cc} s_{k-1}+\alpha_{\text{up}}\left({\hat{y}}_{k}-s_{k-1}\right),\; & \text{if }{\hat{y}}_{k}>s_{k-1},\;0<\alpha_{\text{up}}<1,\\ s_{k-1}+\alpha_{\text{down}}\left({\hat{y}}_{k}-s_{k-1}\right),\; & \text{if }{\hat{y}}_{k}\leq s_{k-1},\;0<\alpha_{\text{down}}\leq 1 \end{array}\right.$$ (2)

Pilot testing determined appropriate baseline values of $\alpha_{\text{up}}=0.065$ (resulting in a rise-time constant of approximately 300 ms at 50 Hz) and $\alpha_{\text{down}}=1$ (allowing immediate reductions). This configuration effectively prevented excessive overshoot while maintaining responsiveness during deactivation.

The filtered scalar output $s_{k}\in \left[0,60\right]\;Nm/rad$ directly set the proportional stiffness term $\left(k_{p}=s_{k}\right)$ in the impedance control law (Equation 3):

$$\tau =k_{p}\left(q_{d}-q\right)+k_{d}\left({\dot{q}}_{d}-\dot{q}\right),$$ (3)

where damping was held constant at $k_{d}=0.3\;Nm\cdot s\cdot rad^{-1}$ to minimize oscillations. Desired joint position $q_{d}$ and velocity ${\dot{q}}_{d}$ targets were zero (upright steady stance $q_{d}={\dot{q}}_{d}=0$ ), ensuring assistance torque depended solely on the displacement $\left(q_{d}-q\right)$ , which not only assists rising, but also regulates the downward motion.

Figure 2 provides a block diagram of the control loop: the multi-channel EMG envelope is mapped via ridge regression to a scalar effort estimate $\hat{y}$ , which is then processed by the slew-rate limiter to yield $s_{k}$ . This signal directly sets the proportional stiffness gain $k_{p}$ , while the desired joint position and velocity remain at zero (upright stance). The resulting impedance law generates a desired torque that is tracked by the low-level current controller of the motor.

Instead of directly commanding joint torque, we modulated joint stiffness $k_{p}$ using impedance control. This approach conceptualizes the joint as a virtual spring-damper system, enabling the actuator to absorb contact forces while remaining passively back-drivable. Such behavior mirrors human neuromotor control strategies, where mechanical impedance, particularly stiffness and damping, is dynamically adjusted to stabilize movements and counteract destabilizing forces (Abu-Dakka and Saveriano, 2020; Burdet et al., 2001).

Although impedance control principles are often applied in complex rehabilitation exoskeletons for precise trajectory tracking (Liu et al., 2021), their benefits extend to simpler, single-degree-of-freedom devices such as our knee orthosis. Scaling stiffness rather than directly prescribing torque maintains high compliance, allowing users to adapt their motion paths while intuitively. Increased stiffness during significant deviations provides a biomechanically secure safety margin, effectively minimizing abrupt torque spikes and large impact forces (Abu-Dakka and Saveriano, 2020; Liu et al., 2021), accommodating inter-individual variability in strength and biomechanics, and closely aligning robotic assistance with natural human muscle stiffness modulation Zhu et al. (2025). For a representative Ortho-ON trial, Supplementary Figure S5 visualizes how the right-thigh EMG envelope and the resulting prediction $\hat{y}$ command the motor torque over the four phases of one full motion cycle.

### 2.3 Experimental setup

Ten able-bodied adults (*age* = 34.5±13.8 y (4:female; 6:male), *height* = 171.9±8.2 cm, *weight* = 70.8±10.4 kg , *BMI* = $23.8\pm 2.2\;kg.\;m^{-2}$ ) were recruited. Exclusion criteria for this study included: untreated injury, neurological disorders, and cardiovascular contraindications. All volunteers provided written informed consent, and the study was approved by the Friedrich-Alexander Universität ethics board (Ref.23–350-B).

**FIGURE 3:**
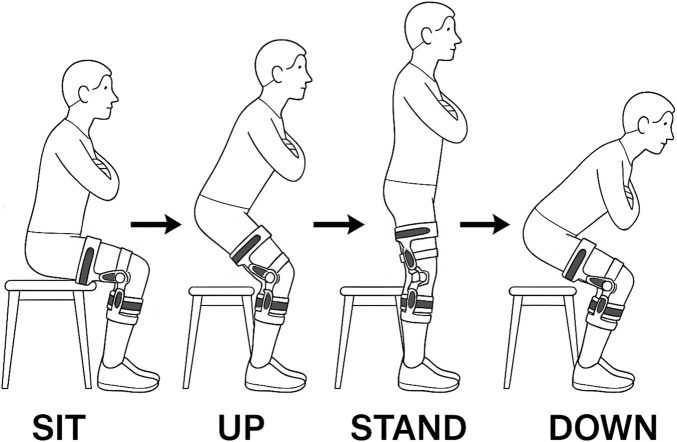
Sit-To-Stand Cycle with illustration of each phase. Arms crossed in front of torso. Orthosis worn on the right side.

Participants executed a Sit-to-Stand ⇌ Stand-to-Sit paradigm and returned to the starting position as displayed in Figure 3 under three successive conditions: *No-Ortho, Ortho-OFF, and Ortho-ON*.

- *No-Ortho*: baseline condition without the orthosis, representing the participant’s undisturbed knee biomechanics.
- *Ortho-OFF*: orthosis worn, actuators unpowered; a torque compensation routine nulls static friction, leaving only the added mass and residual joint stiffness of the device.
- *Ortho-ON*: active measurement condition in which the orthosis delivers EMG-driven assistance. The proportional stiffness of the impedance controller is set to $k_{p}=s_{k}$ , while the damping coefficient is held constant at $k_{d}=0.3\;Nm\cdot rad^{-1}$ to prevent oscillations.

For every condition, each subject performed 15 uninterrupted STS cycles with their arms crossed in front of their chest on a rigid, armless chair (seat height: 47 cm). The order of conditions was fixed (No-Ortho → Ortho-OFF → Ortho-ON) to gradually introduce the device; consequently, potential learning or fatigue across blocks cannot be ruled out and is reported as a limitation. A digital metronome set to 20 beats per minute (20 bpm: 3 s inter-beat interval) provided auditory cues. At each beat, the participant was instructed to initiate either the concentric (UP) or eccentric (DOWN) transfer, depending on the preceding stable resting state (SIT or STAND). The four resulting phases are thus defined and cycled through as SIT → UP → STAND → DOWN (Schenkman et al., 1990).

The two baseline measurements (*No-Ortho, Ortho-OFF*) allow quantification of natural performance and the passive mechanical burden introduced by the device, respectively; these serve as references against which the EMG-assisted *Ortho-ON* condition is evaluated.

### 2.4 Outcome measures and statistical analysis

#### 2.4.1 EMG postprocessing and feature rationale

To isolate orthosis effects on muscular activation across the conditions (*No-Ortho, Ortho-OFF, Ortho-ON*) and movement phases, offline EMG signals were high-pass filtered at 20 Hz to minimize motion artifacts while preserving the relevant EMG spectrum. Power-line interference was eliminated using a 50 Hz notch filter (Boyer et al., 2023). Subsequently, each channel was z-normalized for inter-subject comparability, rectified, and smoothed using a 50 ms sliding window to compute the ARV (offline). ARV features were selected due to its direct correlation with motor unit recruitment and muscle effort (De Luca, 1997; Zhou and Rymer, 2004), and its robustness compared to root mean square (RMS), particularly regarding sensitivity to outliers and non-Gaussian amplitude distributions (Clancy and Hogan, 1997). Lower median ARV features values indicate reduced muscular effort, while decreased ARV features variance denotes enhanced neuromuscular stability and efficient force production (Goubault et al., 2023).

#### 2.4.2 Biomechanical threshold phase segmentation

Phase boundaries were identified using a rule-based approach integrating thresholds derived from biomechanical findings by Kralj et al. (1990) and Norman-Gerum and McPhee (2020). The seat-off transition (SIT → UP) was marked when knee flexion angle $\left(\theta_{k}\right)$ decreased below 85° , knee extension velocity $\left({\dot{\theta }}_{k}\right)$ exceeded $10{}^{\circ}\cdot s^{-1}$ , and horizontal COM velocity surpassed $0.05\;m\cdot s^{-1}$ . The STAND phase was declared once the knee angle fell below 5° and absolute vertical COM velocity dropped below $0.02\;m\cdot s^{-1}$ . The DOWN phase onset was determined when downward COM acceleration $\left(a_{\text{COM},z}\right)$ dropped below $-0.25\;m\cdot s^{-2}$ or knee flexion again surpassed 8° . The DOWN phase ended when the knee angle returned close to the resting position $\left(\theta_{k}\approx 90{}^{\circ}\pm 5{}^{\circ}\right)$ .

#### 2.4.3 Outcome metrics and statistical analysis

For each sit-to-stand repetition, we computed median values, variance, and the 95th percentile $\left(q_{95}\right)$ of the average rectified value (ARV) distribution to quantify muscular activation. Effect sizes between conditions were assessed using Hedges’ g , applying small-sample bias correction. Left and right extensor muscle ARVs were spatially averaged via the median and analyzed both separately and as a combined bilateral measure $\text{ARV}=\frac{1}{2}\left({\text{ARV}}_{L}+{\text{ARV}}_{R}\right)$ .

Given the strictly positive and right-skewed nature of ARV data, a natural log transform was applied $\left(y=\ln\left(\text{ARV}+\epsilon \right),\;\epsilon =10^{-6}\right)$ to stabilize variance and reduce skewness, a widely accepted practice for physiological data (West, 2022). Linear mixed-effects models (LMMs) were then fitted separately for each distinct movement phase (SIT, UP, STAND, DOWN), reflecting the biomechanical differences inherent to each sub-phase (Herzog et al., 2025).

For a given phase j , the LMM was structured as follows:

$$y_{ik\ell }=\beta_{0}+\beta_{\text{Cond}}{\text{Cond}}_{i}+\beta_{\text{Trend}}{\text{TrialNr}}_{\ell }+\left(1|{\text{ID}}_{k}\right)+\varepsilon_{ik\ell },$$

where ${\text{Cond}}_{i}\in \left\{\text{No},\text{OFF},\;\text{ON}\right\}$ represented orthosis states, dummy-coded with *No Ortho* as the reference category. In addition to the fixed factor *Condition*, a linear trial progression term ( ${\text{TrialNr}}_{\ell }$ = 1 … 15) was included to capture systematic learning or fatigue effects within each block, accounting for the fixed condition order. A random intercept $\left(1|{\text{ID}}_{k}\right)$ was included to capture participant-specific variability.

Three planned contrasts (ON vs. No, OFF vs. No, ON vs. OFF) were extracted from fixed effects for each phase. Back-transformed estimates from the log scale provided interpretable percent changes $\left(\Delta \%=100\;\left[\exp\left(\hat{\beta }\right)-1\right]\right)$ , accompanied by 95% Wald confidence intervals. To maintain a family-wise error rate of α=0.05 across the twelve tests (four phases, three contrasts each), Bonferroni correction was applied (adjusted $p_{\mathrm{adj}}=\min\left\{p\times 12,1\right\}$ ), with $p_{\mathrm{adj}}<0.05$ considered significant with *p_adj_/p<0.05; **p_adj_/p<0.01; ***p_adj_/p<0.001 (Bland and Altman, 1995).

To examine the orthosis’s influence on activation stability, the same analysis pipeline was applied to the log-transformed trial-wise variance of ARV, aggregated by participant and condition (Pinheiro, 2002). Model diagnostics included checking residual plots to ensure homoscedasticity and normality assumptions were met.

#### 2.4.4 Questionnaire and NASA-TLX

The participants were given questionnaires to complete before and after the exercises. The Pre-Study questionnaire evaluates the frequency of orthotic, robotic, and EMG use, as well as the frequency and type of exercise, categorized into “Endurance and Cardiovascular”, “Strength and Fitness”, “Team”, “Racket and Precision”, “Adventure and Action”, and “Mind-Body and Movement”. After performing each condition, the NASA Task Load Index (NASA-TLX) questionnaire was used to assess the subjective mental workload (Hart and Staveland, 1988). The questionnaire contains six criteria: Mental Demand (MD), Physical Demand (PD), Temporal Demand (TD), Performance (P), Effort (E), and Frustration (F). Each NASA-TLX subscale ranges from 0 (low demand) to 20 (high demand); weighted scores were computed on a 0–100 scale, with higher values indicating greater perceived workload. In our reporting, higher scores therefore reflect increased subjective burden. In addition to the NASA-TLX questionnaire, the participants received supplementary post-study Likert-style question items regarding perception of system usability, task complexity, comfort, and the effectiveness of feedback during orthosis interaction.

## 3 Results

### 3.1 EMG activation and orthosis effectiveness

**FIGURE 4:**
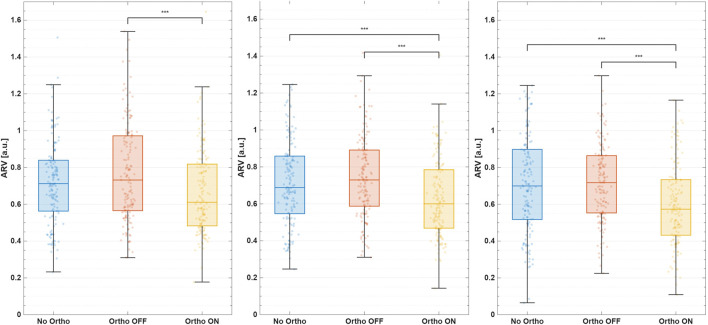
Median ARV during the UP phase. From left to right: left leg, bilateral mean, right leg.

**FIGURE 5:**
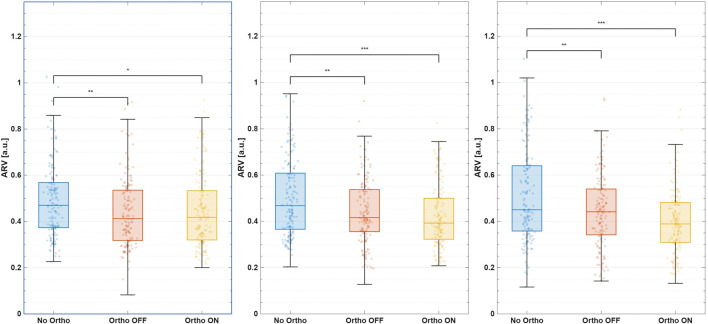
Median ARV during the DOWN phase. From left to right: left leg, bilateral mean, right leg.

Table 1 summarizes phase-specific changes in median bilateral log-ARV. Side-specific responses (left and right legs) and variance analyses are detailed in the Supplementary Material, Supplementary Tables S1–S5. Medians were reported to minimize sensitivity to outliers, with dispersion visualized through boxplots (Figures 4, 5).

**TABLE 1:** Percentage change in bilateral ARV activity (mean of left + right) for each movement phase. Effects that survive the family-wise criterion $p_{\mathrm{adj}}<0.05$ are bold. *p_adj_/p<0.05; **p_adj_/p<0.01; ***p_adj_/p<0.001.

Phase	Contrast	Δ%	95% CI	g	p	$p_{\mathrm{adj}}$
SIT	**ON–No**	**−11.7**	**[-16.5, -6.7]**	**−4.4**	<0.001	${<0.001}^{***}$
	OFF–No	−5.7	[-10.8, −0.4]	−2.1	0.038	0.452
	ON–OFF	−6.3	[-11.4, −1.0]	−2.3	0.021	0.249
UP	**ON–No**	**−11.2**	**[-15.8, -6.4]**	**−4.4**	<0.001	${<0.001}^{***}$
	OFF–No	5.7	[ 0.3, 11.5]	2.1	0.040	0.475
	**ON–OFF**	**−16.0**	**[-20.4, -11.5]**	**−6.5**	<0.001	${<0.001}^{***}$
STAND	ON–No	3.1	[-4.3, 11.0]	0.8	0.425	1.000
	**OFF–No**	**13.2**	**[5.1, 21.0]**	**3.3**	<0.01	${<0.01}^{**}$
	ON–OFF	−8.9	[-15.5, −1.9]	−2.5	<0.05	0.170
DOWN	**ON–No**	**−15.4**	**[-20.6, -9.8]**	**−5.2**	<0.001	${<0.001}^{***}$
	**OFF–No**	**−12.1**	**[-17.5, -6.3]**	**−4.0**	<0.001	${<0.01}^{**}$
	ON–OFF	−3.74	[-9.7, 2.6]	−1.2	0.242	1

#### 3.1.1 UP-phase (sit-to-stand ascent)

Active assistance reduced bilateral extensor activation by −11.2% relative to *No-Ortho* and −16.0% relative to *Ortho-OFF* (both $p_{\mathrm{adj}}<0.001$ , effect size |g|≈4.4 – 6.5 , see Figure 5). Relative to the *No-Ortho* condition, *Ortho-ON* assistance reduced median extensor ARV on the braced right leg by −14.8% ( $p_{\mathrm{adj}}<0.001$ , |g|=5.2 ) during ascent, whereas the unbraced left leg showed a smaller decrease of −7.2% (Supplementary Material: Supplementary Tables S1, S2). This combination corresponds to an ≈8% reduction in the right-to-left ARV ratio ( ${\text{ARV}}_{R}$ / ${ARV}_{L}$ ) in the UP phase, indicating that the assisted limb is unloaded more than the contralateral limb without evidence of compensatory over-recruitment. While variance reduction initially appeared sub-threshold ( −28.2% , $p_{\mathrm{adj}}=0.13$ ), combined analysis revealed considerable bilateral variance reductions, indicating improved muscular activation consistency and movement control (Supplementary Material: Supplementary Table S3, Supplementary Figure S1).

#### 3.1.2 DOWN-phase (*stand-to-sit descent*)

During descent, the active orthosis significantly lowered bilateral median ARV by −15.4% compared to *No-Ortho* ( $p_{\mathrm{adj}}<0.001$ , |g|=5.2 ), and −12.1% relative to *Ortho-OFF* ( $p_{\mathrm{adj}}<0.01$ , |g|=4.0 ). During the eccentric DOWN phase, *Ortho-ON* assistance reduced median ARV on the braced right leg by −20.7% ( $p_{\mathrm{adj}}<0.001$ , |g|=6.2 ) relative to *No-Ortho*, whereas the left leg decreased by −10.0% (Supplementary Material: Supplementary Tables S1, S2). This corresponds to an ≈12% reduction in the right-to-left ARV ratio compared with *No-Ortho*, again suggesting preferential unloading of the assisted limb rather than contralateral overuse. For the right (braced) leg, ARV variance during the DOWN phase decreased significantly by −44% ( $p_{\mathrm{adj}}<0.05,\;|g|=3.2$ ; Supplementary Tables S3–S5; Supplementary Figure S2), indicating more repeatable muscle activation across repetitions.

#### 3.1.3 Static phases (SIT and STAND)

During SIT, the active orthosis significantly reduced bilateral median ARV by −11.7% ( $p_{\mathrm{adj}}<0.001$ , |g|=4.4 ), primarily driven by a pronounced decrease on the braced leg ( −20.5% , $p_{\mathrm{adj}}<0.001$ ). Variance reductions remained non-significant. In the STAND phase, no significant activation reductions occurred. However, the unpowered orthosis condition caused a slight but significant bilateral ARV increase ( 13.2% , $p_{\mathrm{adj}}<0.01$ ), suggesting minor compensatory activation due to device mass.

#### 3.1.4 Within-session adaptation

##### 3.1.4.1 ARV learning over time

**FIGURE 6:**
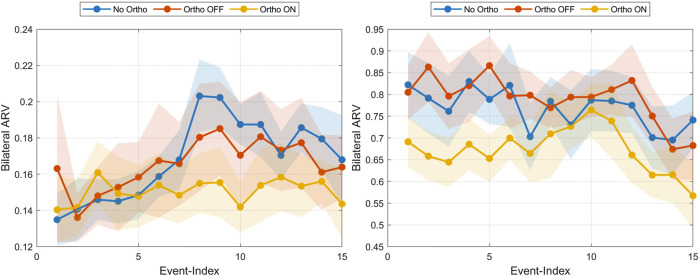
Median bilateral ARVs averaged over all participants (mean±SEM) across all trials for the SIT phase (left) and UP phase (right) visualized for each condition.

Median bilateral quadriceps ARV showed only modest, statistically non-significant within-session changes across repetitions (Figure 6; Table 2). During the preparatory SIT phase, ARV gradually increased by 3.6% on average $\left(p_{a}dj=0.134\right)$ , which may reflect weak anticipatory priming response upon auditory cues for the next anticipated movement. In contrast, concentric UP-phase ARV decreased by −5.7% $\left(p_{a}dj=0.635\right)$ , consistent with a trend toward more economical use of the assistance, but these changes did not reach statistical significance and should therefore be interpreted as exploratory. These adaptations emerged despite no prior device familiarization.

**TABLE 2:** Pooled change in ARV from early (trials 1–5) to late (trials 11–15); Epoch × Condition interaction was non-significant, therefore the values are averaged across the Orthosis Conditions No, OFF, and ON.

	Phase	Δ%	$p_{\mathrm{adj}}$
$q_{95}$	SIT	+5.2	1
	UP	−13.4	0.546
	STAND	+0.4	1
	DOWN	−5.0	1
Median	SIT	+3.6	0.134
	UP	−5.7	0.635
	STAND	−2.7	0.804
	DOWN	−1.5	1

##### 3.1.4.2 Torque learning over time

**FIGURE 7:**
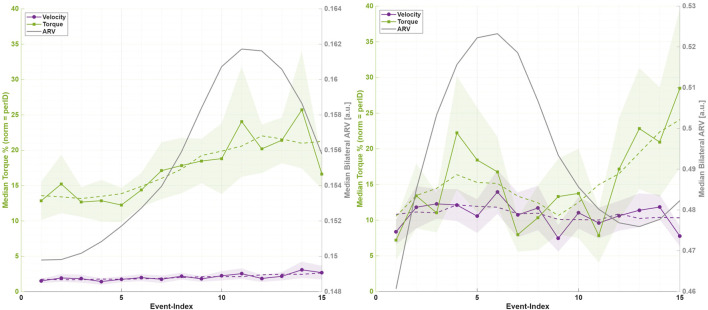
Median orthosis torque output normalized per participant (mean±SEM) across all trial events for the SIT phase (left) and DOWN phase (right). The dark overlay curve shows the median bilateral ARV.

Torque analysis revealed significant within-session adaptations (Table 3; Figure 7). During SIT, participants significantly increased anticipatory torque engagement ( Δ=0.084 , d=0.94 , p<0.05 ), aligning with the ARV priming observed above. Moderate positive correlations between torque and ARV ( r=0.30 , p<0.001 ) underscored a preparatory strategy, enabling more efficient and synchronized orthosis utilization upon movement initiation. In contrast, the UP phase displayed stable torque demands, indicating rapid establishment of movement patterns without further significant adaptation. The eccentric DOWN phase showed slight, non-significant torque increases, moderately correlated with declining ARV ( r=0.41 , p<0.001 ), reflecting more controlled eccentric activation.

**TABLE 3:** Early-late change (trials 1–5 vs. 11–15) and Pearson correlation with bilateral ARV by phase. Values per-ID normalized $\left(x\cdot \max|x{|}^{-1}\right)$ . p : uncorrected; effect: Cohen’s d . *p_adj_/p<0.05; **p_adj_/p<0.01; ***p_adj_/p<0.001.

Phase	Signal	Δ	Test	p	d	$r_{\text{ARV}}$	$p_{\text{ARV}}$
SIT	Velocity	0.008	t(9)=1.51	0.165	0.5	0.233	<0.01
SIT	Torque	0.084	t(9)=2.98	${<0.05}^{*}$	0.9	0.296	<0.001 ***
UP	Velocity	0.013	t(9)=1.65	0.133	0.5	0.267	<0.001
UP	Torque	0.003	t(9)=0.07	0.944	0.0	0.060	0.465
STAND	Velocity	0.000	W=22	0.625	—	−0.190	<0.05
STAND	Torque	0.000	t(9)=0.21	0.838	0.1	−0.017	0.837
DOWN	Velocity	−0.008	t(9)=−1.44	0.184	−0.5	0.452	<0.001
DOWN	Torque	0.018	W=40	0.232	—	0.408	<0.001

##### 3.1.4.3 Participant utilization of orthosis power

**FIGURE 8:**
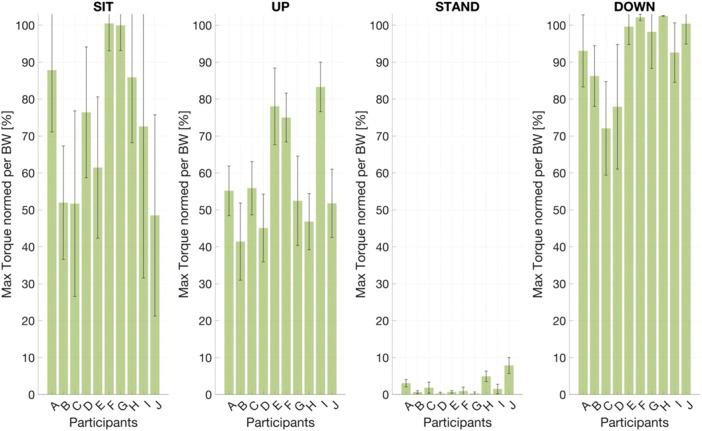
Capitalization on orthosis power output per participant. Columns show the power facilitated by each participant (mean±SD) relative to the maximum possible achievable output.

Peak orthosis power utilization varied by movement phase (Figure 8). Highest torque engagement occurred during eccentric descent (DOWN), averaging 83±22% of the maximum capacity. Conversely, concentric UP engagement was modest (66±10%) , reflecting either limited familiarity or conservative initial lifting strategies. During preparatory SIT, participants displayed mixed strategies. Approximately half of the participants utilized the provided torque capacity, while a few used only 50% . The group-level relationship in the active Ortho-ON condition between right-leg EMG ARV and the commanded stiffness gain $k_{p}=s_{k}$ is shown in Supplementary Figure S3. The commanded stiffness increases from single-digit values at low activation to approximately $20-30\;Nm\cdot rad^{-1}$ at moderate right-leg ARV. Additionally, Supplementary Figure S4 depicts the corresponding parabolic group-averaged torque-angle trajectory. Participants reach maximum torque support midway through the motion, with noisier responses at maximum deflection angles.

### 3.2 Subjective measurements: questionnaires and NASA-TLX

Participants predominantly had limited prior exposure to orthoses and EMG systems (Supplementary Material: Supplementary Table S6). Exercise frequency was high, primarily endurance-based, reflecting a physically active cohort with minimal specialized robotics experience.

**FIGURE 9:**
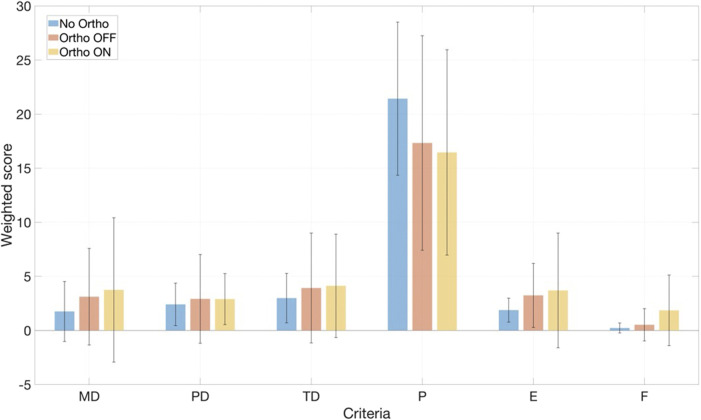
Results of the NASA-TLX weighted workload scores (mean±SD) across all tested conditions.

NASA-TLX results (Figure 9) indicated modest overall workload without significant differences between conditions (Wilcoxon signed-rank tests, all p>0.05 ). Performance ratings were consistently highest but showed a downward trend with orthosis use, indicating minimal perceived disruption by the device.

Participants rated orthosis usability positively (75–79 mean scores), with intuitive control perceptions remaining stable. Despite minor decreases in intuitiveness under active conditions, overall task complexity remained low. Visual and acoustic feedback was highly valued (82–87 mean scores), with a slight preference for more device-generated feedback over supervisor cues. Discomfort remained moderate, with slight increases during active power, reflecting acceptable comfort levels.

## 4 Discussion

Powered assistance significantly reduced bilateral median EMG activity during both ascent and descent phases, even after Bonferroni correction. Additionally, the muscle activity reduction during ascent remained significant when comparing the powered orthosis to its unpowered condition. Another important finding was a notable decrease in the variability of right-leg muscle activity during descent, though the bilateral variance decrease did not remain significant after correction. Taken together, these findings indicate that the powered orthosis effectively eased muscular effort during the primary movement phases and promoted more consistent muscle activation patterns, particularly for the supported leg during controlled descent.

### 4.1 Interpretation of EMG and variance results

These findings align well with the intended function of an EMG-proportional impedance controller, which modulates assistance based on measured muscle activation. The observed lower median EMG amplitudes under powered conditions suggest that the orthosis successfully replaced part of the biological muscle torque, effectively reducing the muscular effort required by users. Side-specific analyses support this interpretation. Across sit, ascent, and descent, the braced right leg consistently showed larger ARV reductions than the unbraced left leg (UP: −14.8% vs. −7.2% ; DOWN: −20.7% vs. −10.0% ; Supplementary Material: Supplementary Tables S1, S2), which implies an ≈8% and ≈12% reduction, respectively, in the right-to-left ARV ratio compared with *No-Ortho*. Within the limits of EMG as a proxy for neuromuscular effort, this pattern indicates preferential unloading of the assisted limb without contralateral overuse. Because no ground-reaction forces or centre-of-pressure data were collected, these EMG-based symmetry measures reflect relative effort redistribution rather than precise mechanical load sharing between limbs. Although the reduced variance in the right leg during descent points toward more consistent muscle activation patterns, this finding alone does not definitively indicate improved stability or movement quality. Confirming stability improvements would require additional kinematic or kinetic evidence, such as detailed joint angles, segment velocities, or ground reaction forces. Consequently, EMG variance should be viewed as descriptive and indicative of neuromuscular behavior, rather than as a direct measure of functional stability or control quality.

The side-specific results further argue against contralateral compensation. The braced right leg exhibited reduced activation during sit, ascent, and descent under powered conditions. Notably, the left leg did not show a compensatory increase. Instead, it demonstrated reduced activation during ascent (powered vs. unpowered) and descent (powered vs. baseline). This consistent bilateral pattern indicates that assistance did not shift demand to the contralateral limb. Additionally, the left-leg reduction during descent suggests bilateral unloading effects, likely due to interlimb coordination rather than direct mechanical support.

### 4.2 Device mechanics and task demands

Phase-specific contrasts shed light on how device mechanics interact with varying task demands. During the stand phase, the unpowered orthosis significantly increased bilateral EMG compared to baseline, an effect that active powering of the orthosis successfully mitigated. This suggests that the unpowered condition introduced additional physical demands, likely due to factors such as device weight, residual friction, or alignment constraints, which the powered assistance partially offset. In contrast, during the sit phase, the powered orthosis significantly reduced bilateral EMG, highlighting that its supportive effects extended beyond dynamic movements alone. Furthermore, during descent, both powered and unpowered conditions resulted in decreased bilateral EMG compared to baseline, with the powered condition providing a notably greater reduction. These observations underline the orthosis’s role in effectively supporting muscular effort across various phases and tasks.

### 4.3 Exploratory analyses: adaptation and power utilization

Exploratory analyses offered further insights into user adaptation patterns and design implications. We observed a significant increase in within-session torque during the sit phase (Δ=0.084,t(9)=2.98,p<0.05) , although median bilateral EMG drifts remained small and statistically insignificant during both sit $\left(+3.6\%,p_{\text{adj}}=0.134\right)$ and ascent $\left(-5.7\%,p_{\text{adj}}=0.635\right)$ . These findings suggest modest adaptation by users across repeated trials, though notable EMG-level changes within sessions were not clearly apparent. Orthosis power utilization was substantially, 73.7±19.4% during descent and 58.5±8.6% during ascent, indicating effective engagement of the actuator. Given the device’s torque ceiling of 16 Nm , it is certain that saturation occurred during more demanding descent phases, potentially limiting further muscular unloading. Consequently, enhancing the device’s torque density emerges as an important consideration for future orthosis design. While the protocol comprised only 15 repetitions per condition, the early-late comparisons and trial-wise trends (Table 2; Figures 6, 7) provide some insight into short-term adaptation. Participants increased anticipatory torque during SIT but showed only small, non-significant drifts in median ARV across repetitions. This pattern is more consistent with strategic adjustment to the EMG-driven assistance than with pronounced fatigue. However, the short exposure and healthy cohort preclude strong conclusions about long-term robustness or training effects. Multi-session protocols, ideally in clinical populations, will be needed to quantify how muscle fatigue and motor adaptation evolve over days or weeks of use.

### 4.4 Subjective workload assessment

Subjective workload, assessed using the NASA-TLX, showed no significant differences between conditions across all subscales (Figure 9). Given the small sample size and the short, highly structured protocol, the study was underpowered to detect potentially subtle subjective benefits such as reduced perceived effort during the dynamic phases. At the same time, the absence of increased workload in the Ortho-ON condition is encouraging, suggesting that the EMG-driven impedance controller can reduce extensor EMG without introducing a noticeable cognitive or physical burden. We expect that longer, more ecological usage scenarios (e.g., repeated STS as part of daily activities or rehabilitation sessions) may reveal clearer subjective benefits, particularly for users with pronounced extensor weakness.

### 4.5 Comparison to recent EMG-controlled knee exoskeletons

To place our findings in the context of recent EMG-controlled knee exoskeleton work, we next compare our results with those of Gunnell et al. (2025). This research reported significant reductions in peak quadriceps EMG (32%) and substantial increases in knee extension torque (59%) in stroke survivors using an EMG-controlled powered knee exoskeleton with a torque limit of $0.5\;Nm\cdot \;kg^{-1}$ . In our healthy cohort, we observed more modest reductions in median ARV (11%–21% depending on phase and leg), which was not always fully exploited during ascent (mean utilization ≈59% ; Figure 7), with a device whose nominal torque capacity $\approx 0.23\;Nm\cdot \;kg^{-1}$ ) is roughly half of that used by Gunnell et al. (2025). This is in line with their research, which achieved a ≈32% reduction in peak quadriceps EMG. Together, these findings suggest a roughly dose-dependent relationship between available assistive torque and achievable EMG reduction, modulated by user adaptation and task demands. Moreover, Gunnell et al. (2025) employed high-frequency EMG sampled at 2000 Hz and targeted a single paretic muscle, whereas our controller relies on consumer-grade dry electrodes at 200 Hz and a global thigh activation pattern. This difference likely affects the precision of activation timing and amplitude estimates. Nevertheless, both studies consistently show that proportional EMG control can reduce extensor effort without inducing detrimental compensations. Our results extend this evidence to a low-cost, minimally calibrated brace, indicating that meaningful unloading may be achievable even with reduced torque capacity and simpler sensing hardware, rather than competing with high-torque custom-built braces.

### 4.6 Study limitations

The fixed order of testing conditions introduces potential sequence or learning effects, possibly influencing within-session comparisons. To mitigate this risk analytically, we explicitly modeled trial progression in the linear mixed-effects models and contrasted early versus late repetitions (Table 2). While modest adaptations were observed, such as increased preparatory ARV and torque in the SIT phase, these changes did not differentially affect any orthosis condition, and Condition × Epoch interactions remained non-significant. Nevertheless, future studies should adopt counterbalanced or randomized designs to decouple device effects from sequence-related adaptation or fatigue fully.

Although consumer-grade dry-electrode hardware simplifies deployment, it is more susceptible to motion artefacts, electrode shift, and sweat-induced impedance changes than laboratory-grade adhesive EMG systems. Yet our choice of sensors aligns with the orthosis’s objective of low cost and practicality. Additionally, our ridge-regression controller and the coarse, single-DoF task reduced the impact of such noise, but long-term robustness under daily-life conditions remains to be demonstrated. Again, here we anticipate that the quick, easy calibration allows immediate updates to the regression model weights when needed.

A further limitation concerns the specificity of the EMG-driven control signal. Because the Myo band spans the anterolateral to anteromedial thigh, the recorded envelopes primarily reflect global quadriceps activity, but inevitably also contain cross-talk from biarticular muscles such as *rectus femoris* and from adjacent hip musculature. Consequently, $\hat{y}$ is best interpreted as a single-leg, sagittal-plane activation measure rather than a pure knee-extensor channel. For the paced STS task studied, where both legs act in phase and extensor demand on the braced limb dominates, a global signal is appropriate and has also been used successfully other studies (Lyu et al., 2019). In more dynamic, cyclic tasks such as walking, quadriceps and hamstring muscles contribute in a phase-dependent manner to both hip and knee motion (Mohammadyari Gharehbolagh et al., 2023; Akl et al., 2021). In those settings, a purely monotonic mapping from a single global thigh signal to knee stiffness would likely be suboptimal. EMG-based gait exoskeletons therefore typically combine muscle-specific EMG features with gait-phase estimation (Chen et al., 2023; de Miguel-Fernández et al., 2023).

Similarly, the orthosis torque ceiling of 16 Nm $\left(\approx 0.23\;Nm\cdot \;kg^{-1}\right)$ may restrict effectiveness for heavier users or faster, more dynamic transitions. The small sample size and healthy cohort constrain statistical precision and generalizability. Given the relatively slow and predictable dynamics of paced STS transfers, the ≈250−300 ms rise time from EMG onset to reaching the 16 Nm torque ceiling did not manifest as perceptible delay or instability, as supported by the absence of abnormal kinematic patterns and the lack of increased subjective workload in the Ortho-ON condition. This is further illustrated by the representative time series for a powered trial (Supplementary Figure S5), where the EMG envelope of the braced thigh rises before the prediction $\hat{y}$ , yet elicited torque and EMG stay largely synced. The temporal smoothing also avoids abrupt torque jumps while still allowing participants to elicit substantial support during an extensor burst UP motion. Together with the high utilisation levels observed during the DOWN phase, this suggests that users quickly learned to exploit the compliant, EMG-scaled stiffness behaviour.

### 4.7 Translational implications and future directions

Our findings provide clear translational implications for future orthosis development. The present prototype still requires integration and is not a plug-and-play consumer product. Our use of an off-the-shelf brace and actuators primarily reduces material cost and facilitates replication by other laboratories, rather than eliminating professional involvement. We therefore frame the device as a low-cost research platform and a potential template for future industrial designs, rather than as an immediately deployable clinical solution. Effective everyday support for sit-to-stand transitions demands higher peak torque capacities without significantly increasing device weight, particularly during the more challenging descent phases. Enhanced mechanical alignment, ergonomics, and design improvements are critical to minimizing the static costs identified in unpowered conditions. Incorporating joint tracking systems, such as inertial measurement units (IMUs), would facilitate continuous joint kinematic monitoring and adaptive state estimation, essential for refining impedance control and enhancing stability assessment. Future studies should adopt randomized or counterbalanced experimental designs and integrate multiple synchronized sensor modalities, including EMG, IMU, and kinetic measurements. Incorporating metabolic and functional outcome assessments will determine whether the EMG reductions observed translate into tangible benefits, such as reduced joint loading, maintained coordination, and lower energy expenditure. Comparative studies against passive braces and other control strategies are necessary to clarify the distinct mechanisms and advantages of active assistance. Additionally, clinical trials involving diverse populations, especially those with motor impairments, are essential to comprehensively evaluate the orthosis’s clinical relevance, usability, and safety. Moreover, the present study evaluated the EMG-driven impedance controller only during paced sit-to-stand transfers in healthy adults. Systematic testing of its versatility across walking, stair ambulation, and everyday transfer tasks, as well as in clinical populations, will be crucial to establish its broader clinical applicability.

## 5 Conclusion

This study provides evidence that a low-cost, EMG-controlled knee orthosis can meaningfully unload the knee extensors during both concentric (UP) and eccentric (DOWN) phases of the sit-to-stand cycle. Powered assistance reduced mean muscle activation on the braced limb without provoking compensatory over-reliance on the contralateral side and without disrupting trial-to-trial activation consistency. Participants rapidly adapted to the impedance controller, entraining anticipatory muscle activity within a single session, while reporting a low perceived workload and acceptable comfort, despite using a first-generation prototype. Torque capacity was limited to 16 Nm ( $\approx 0.23\;Nm\cdot kg^{-1},\approx 15\text{–}57\%$ of typical STS demand), restricting applicability to heavier users or faster, more dynamic tasks. Results stem from short-term exposure in healthy adults. Longitudinal effects, clinical populations, and higher functional loads remain untested. Finally, intent detection relied on a sparse, manually calibrated EMG array that is susceptible to noise and electrode shift.

Our findings demonstrate that a retrofitted CE post-operative knee brace with consumer-grade EMG sensing and minimal calibration can reduce quadriceps effort during sit-to-stand without increasing subjective workload in healthy adults. These results support the technical feasibility of EMG-driven impedance assistance in a low-cost, lightweight form factor. However, we did not evaluate kinematic quality, pain, or functional outcomes in patient populations, and exposure was limited to a short, single-session protocol. We therefore view the present work as an initial step toward using existing orthoses equipped with motor hardware in early post-operative or neurological knee rehabilitation. Future work should focus on increasing motor torque and structural rigidity, while integrating IMU cues for multimodal intent detection, and implementing self-calibrating EMG gain adjustment are immediate priorities. Multi-session trials with post-operative patients will be required to confirm long-term efficacy and to refine controller parameters that balance assistance with progressive neuromuscular challenge.

## Data Availability

The raw data supporting the conclusions of this article will be made available by the authors, without undue reservation.
