# Supplementary material for: EMG-controlled knee orthosis lowers effort in sit-to-stand
Source: Front Robot AI. 2026 Jan 8;12:1732294. doi: 10.3389/frobt.2025.1732294 (PMC12823904; doi:10.3389/frobt.2025.1732294)
Supplement: Supplementary file 1 [file DataSheet1.pdf]

Supplementary Material

0.1 Figures

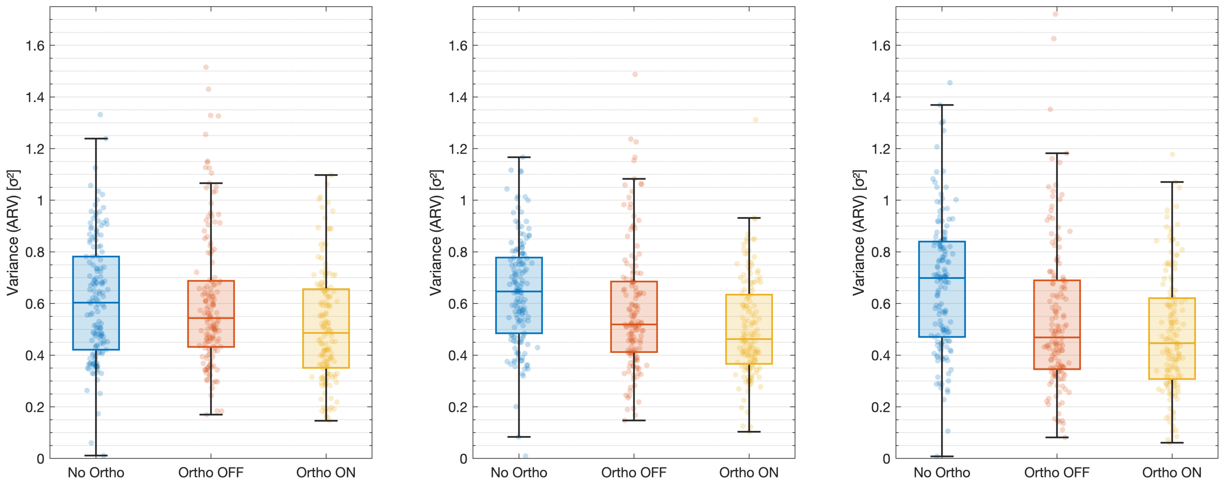

Figure S1. Median ARV Variance during the UP phase. From left to right: left leg, bilateral mean, right leg.

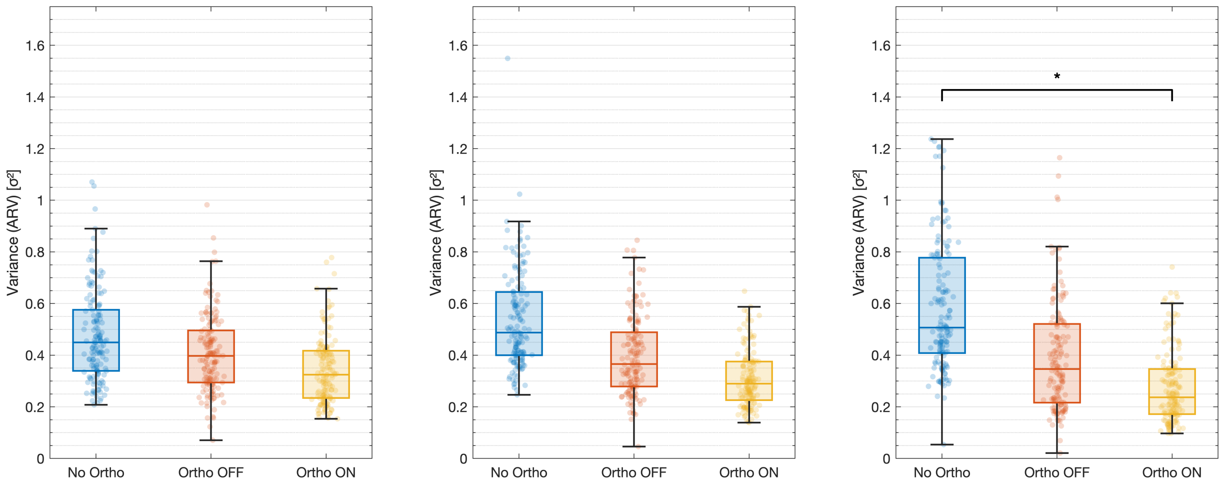

Figure S2. Median ARV Variance during the DOWN phase. From left to right: left leg, bilateral mean, right leg.

0.2 Tables

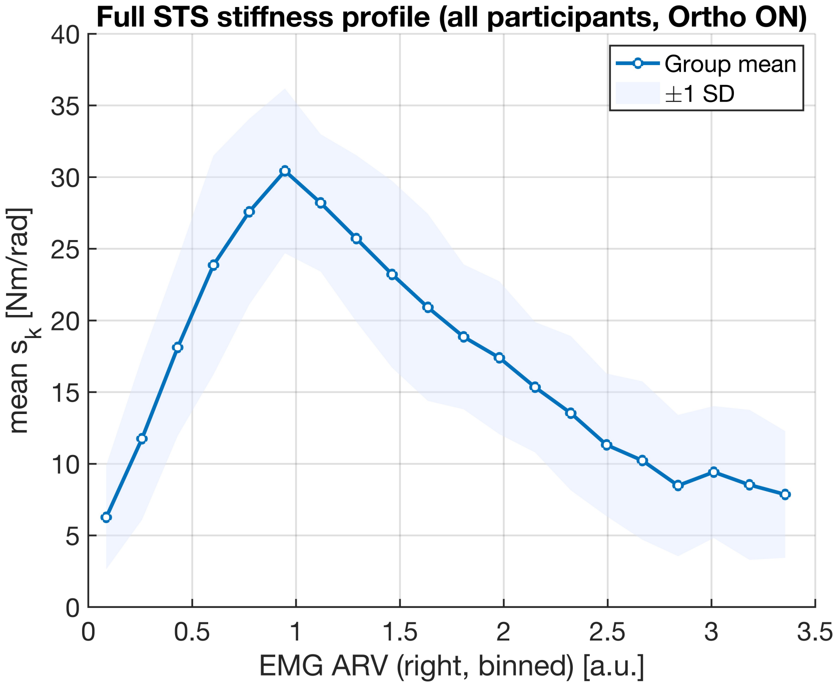

Figure S3. Group-averaged relationship between right-leg ARV and commanded stiffness during Ortho-ON trials.

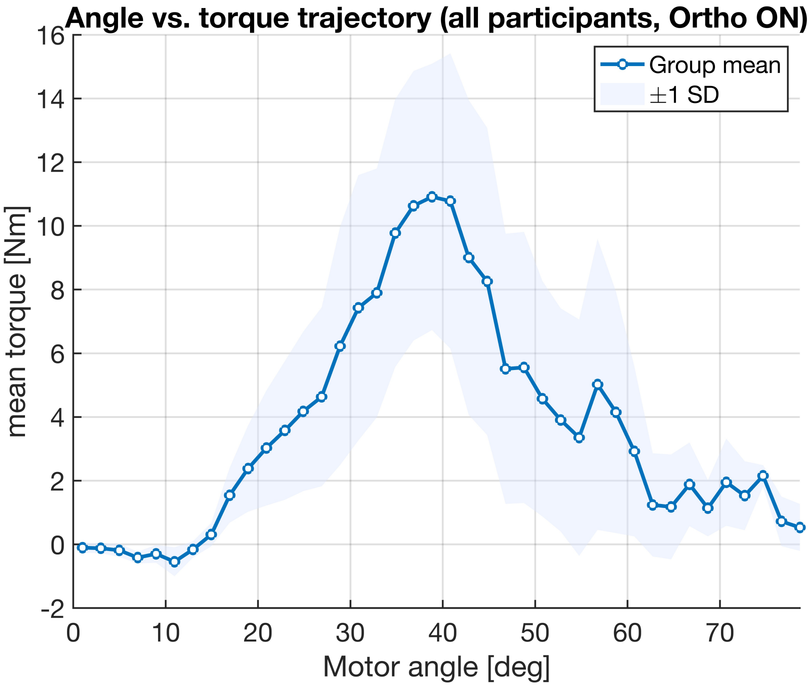

Figure S4. Group-averaged torque-angle trajectory in the Ortho-ON condition.

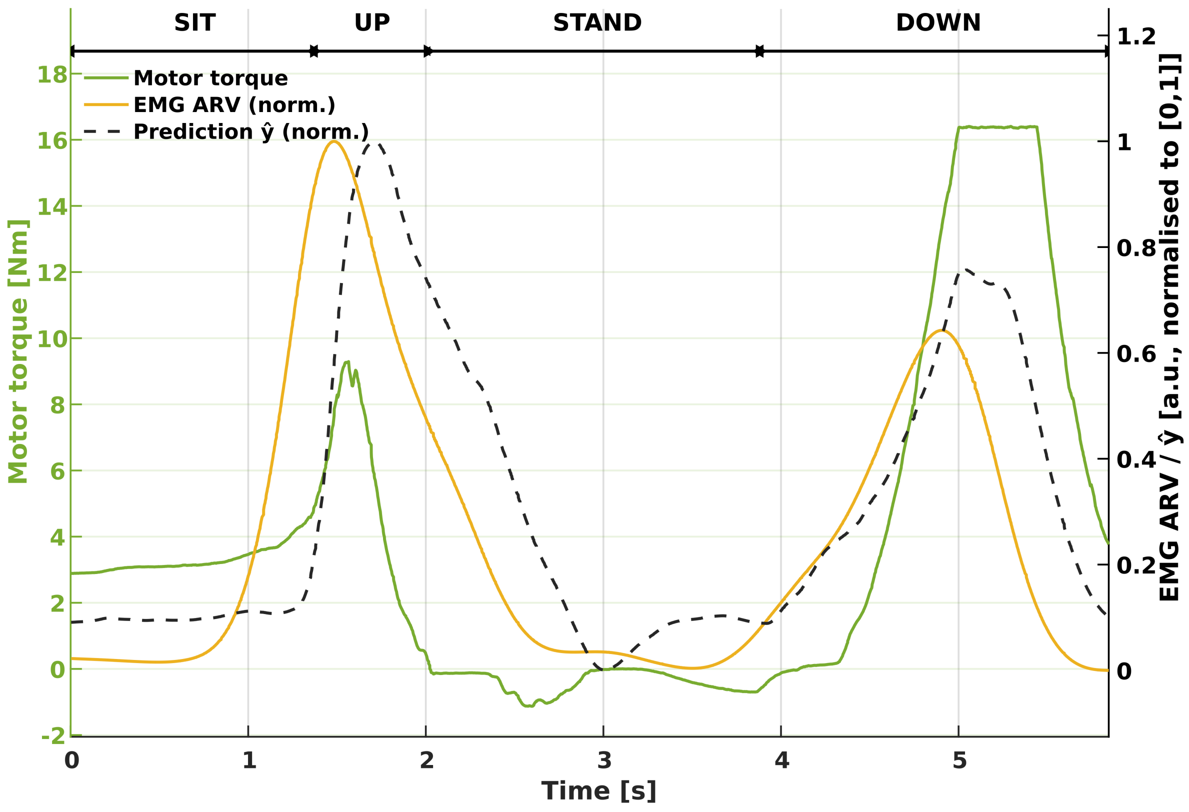

Figure S5. Representative Ortho-ON trial showing motor torque $\tau(t)$, normalized ARV envelope of the braced right thigh, normalized prediction estimate $\hat{y}$ across a full sit-to-stand cycle. Task phases are indicated at the top.

Table S1. Percentage change in log-ARV of the left leg. Values in bold satisfy $p_{adj} < 0.05$.

Phase	Contrast	Δ%	95 % CI	g	p	p_{adj}
	ON–No	1.1	$[-4.8,\ 7.3]$	0.4	0.726	1
SIT	OFF–No	−3.6	$[-9.2,\ 2.3]$	−1.2	0.227	1
	ON–OFF	4.9	$[-1.2, 11.3]$	1.6	0.119	1
	ON–No	-7.2	$[-12.6,\ -1.7]$	-2.5	0.014	0.163
UP	OFF–No	7.0	$[\ 0.8, 13.5]$	2.2	0.028	0.330
	**ON–OFF**	**-13.3**	$\mathbf{[-18.3,\ -8.0]}$	**-4.7**	$\mathbf{< 0.001}$	$\mathbf{< 0.001}^{***}$
	ON–No	-6.4	$[-13.4,\ 1.2]$	-1.7	0.098	1
STAND	OFF–No	-2.1	$[-9.5,\ 5.8]$	-0.5	0.593	1
	ON–OFF	-4.4	$[-11.6,\ 3.4]$	-1.3	0.264	1
	**ON–No**	**-10.0**	$\mathbf{[-15.6,\ -3.9]}$	**-3.2**	$\mathbf{< 0.01}$	$\mathbf{< 0.05}^{*}$
DOWN	**OFF–No**	**-11.4**	$\mathbf{[-17.0,\ -5.5]}$	**-3.6**	$\mathbf{< 0.001}$	$\mathbf{< 0.01}^{**}$
	ON–OFF	1.6	$[-4.8,\ 8.5]$	0.5	0.626	1

Table S2. Percentage change in log-ARV of the right leg. Contrasts with $p_{adj} < 0.05$ are shown in bold.

Phase	Contrast	Δ%	95 % CI	g	p	p_{adj}
SIT	**ON–No**	**-20.5**	**[-26.3, -14.3]**	**-6.0**	**< 0.001**	**$< 0.001^{***}$**
	OFF–No	-5.4	[-12.3, 1.9]	-1.5	0.144	1
	**ON–OFF**	**-15.9**	**[-22.0, -9.4]**	**-4.5**	**< 0.001**	**$< 0.001^{***}$**
UP	**ON–No**	**-14.8**	**[-20.1, -9.1]**	**-4.9**	**< 0.001**	**$< 0.001^{***}$**
	OFF–No	5.6	[-1.0, 12.6]	1.7	0.099	1
	**ON–OFF**	**-19.3**	**[-24.3, -13.9]**	**-6.5**	**< 0.001**	**$< 0.001^{***}$**
STAND	ON–No	9.7	[-1.2, 21.8]	1.7	0.082	0.988
	OFF–No	16.3	[4.8, 29.2]	2.8	< 0.01	0.058
	ON–OFF	-5.7	[-15.1, 4.7]	-1.1	0.274	1
DOWN	**ON–No**	**-20.7**	**[-26.2, -14.7]**	**-6.2**	**< 0.001**	**$< 0.001^{***}$**
	**OFF–No**	**-13.0**	**[-19.1, -6.4]**	**-3.8**	**< 0.001**	**$< 0.01^{**}$**
	ON–OFF	-8.8	[-15.2, -1.9]	-2.5	< 0.05	0.164

Table S3. Percentage change in log-ARV variance (average of left and right leg).

Phase	Contrast	Δ%	95 % CI	g	p	p_{adj}
SIT	ON–No	-29.0	[−51.8, 4.6]	-1.7	0.094	1
	OFF–No	-12.9	[−40.9, 28.1]	-0.7	0.488	1
	ON–OFF	-18.4	[−44.6, 20.1]	-1.0	0.311	1
UP	ON–No	-8.5	[−30.6, 20.7]	-0.6	0.535	1
	OFF–No	-11.5	[−32.8, 16.7]	-0.9	0.396	1
	ON–OFF	3.4	[−21.6, 36.3]	0.2	0.816	1
STAND	ON–No	-17.9	[−39.1, 10.5]	-1.3	0.204	1
	OFF–No	0.2	[−25.6, 35.0]	0.0	0.987	1
	ON–OFF	-18.1	[−39.2, 10.2]	-1.3	0.199	1
DOWN	ON–No	-37.3	[−55.0, −12.5]	-2.8	< 0.05	0.127
	OFF–No	-32.5	[−51.6, −5.8]	-2.3	< 0.05	0.342
	ON–OFF	-7.1	[−33.4, 29.6]	-0.4	0.669	1

Table S4. Percentage change (Δ%) in the variance of log-ARV for the left leg. No contrast survived the corrected threshold $p_{adj} < 0.05$

Phase	Contrast	Δ%	95 % CI	g	p	p_{adj}
SIT	ON–No	-21.9	[−46.9, 14.7]	-1.3	0.219	1
	OFF–No	11.5	[−24.1, 63.9]	0.6	0.583	1
	ON–OFF	-30.0	[−52.4, 2.9]	-1.8	0.081	0.967
UP	ON–No	13.4	[−17.6, 56.1]	0.8	0.447	1
	OFF–No	3.9	[−24.5, 42.9]	0.2	0.817	1
	ON–OFF	9.2	[−20.7, 50.3]	0.5	0.594	1
STAND	ON–No	-10.9	[−36.3, 24.5]	-0.7	0.504	1
	OFF–No	-17.6	[−41.1, 15.1]	-1.1	0.266	1
	ON–OFF	8.2	[−22.6, 51.2]	0.5	0.650	1
DOWN	ON–No	-22.9	[−44.0, 6.2]	-1.6	0.123	1
	OFF–No	-23.3	[−44.3, 5.7]	-1.6	0.116	1
	ON–OFF	0.5	[−27.1, 38.4]	0.0	0.977	1

Table S5. Percentage change ($\Delta\%$) in the variance of log-ARV for the right leg. Contrasts with $p_{adj} < 0.05$ are typeset in bold.

Phase	Contrast	$\Delta\%$	95 % CI	g	p	p_{adj}
SIT	ON–No	-33.3	$[-59.5,\ 9.9]$	-1.6	0.123	1
	OFF–No	-30.2	$[-57.7,\ 15.0]$	-1.4	0.169	1
	ON–OFF	-4.4	$[-42.0,\ 57.5]$	-0.2	0.860	1
UP	ON–No	-28.2	$[-43.3,\ -9.0]$	-2.7	< 0.05	0.131
	OFF–No	-21.7	$[-38.3,\ -0.8]$	-2.1	0.053	0.632
	ON–OFF	-8.2	$[-27.6,\ 16.4]$	-0.7	0.485	1
STAND	ON–No	-7.9	$[-37.8,\ 36.6]$	-0.4	0.686	1
	OFF–No	14.5	$[-22.7,\ 69.7]$	0.7	0.505	1
	ON–OFF	-19.6	$[-45.7,\ 19.3]$	-1.1	0.288	1
DOWN	**ON–No**	**-44.0**	$\mathbf{[-60.9,\ -19.7]}$	**-3.2**	$\mathbf{< 0.01}$	$\mathbf{< 0.05}^*$
	OFF–No	-36.7	$[-55.8,\ -9.2]$	-2.5	< 0.05	0.231
	ON–OFF	-11.5	$[-38.2,\ 26.8]$	-0.7	0.511	1

Table S6. Summary of Participant Pre-Study Survey (n=10) on orthotic, robotic and EMG use, as well as exercise regimen

**Category**	**Subcategory**	**Frequency (%)**
**Orthosis Use**	Never	80% (8)
	Occasional	10% (1)
	Monthly	10% (1)
**Exercise Frequency**	Daily	60% (6)
	Weekly	30% (3)
	Never	10% (1)
**Exercise Type** (Multiple Choice)	Endurance/Cardio	(6)
	Adventure/Action	(3)
	Strength/Fitness	(1)
	Racket/Precision	(1)
	None	(3)
**Robotics Experience**	Never	50% (5)
	Occasional	30% (3)
	Weekly	10% (1)
	Yearly	10% (1)
**EMG Experience**	Never	60% (6)
	Occasional	20% (2)
	Monthly	10% (1)
	Weekly	10% (1)
